# Effect of Ketamine on LTP and NMDAR EPSC in Hippocampus of the Chronic Social Defeat Stress Mice Model of Depression

**DOI:** 10.3389/fnbeh.2018.00229

**Published:** 2018-10-09

**Authors:** Yu Yang, Weina Ju, Haining Zhang, Li Sun

**Affiliations:** Department of Neurology and Neuroscience Center, First Hospital of Jilin University, Changchun, China

**Keywords:** ketamine, depression, LTP, NMDAR, EPSC

## Abstract

Depression is a common mental disorder that is associated with memory dysfunction. Ketamine has recently been demonstrated to be a rapid antidepressant. The mechanisms underlying how depression induces memory dysfunction and how ketamine relieves depressive symptoms remain poorly understood. This work compared three groups of male C57BL/6J mice: mice exposed to chronic social defeat stress (CSDS) to induce a depression-like phenotype, depression-like mice treated with ketamine, and control mice that were not exposed to CSDS or treated with ketamine. Spatial working memory and long term memory were assessed by spontaneous alternation Y-maze and fear conditioning tests, respectively. We used western blot to analyze the density of *N*-methyl-D-aspartate receptor (NMDAR) subunits in the hippocampus. We recorded long term potentiation (LTP) and NMDA receptor-mediated excitatory postsynaptic currents (EPSCs) in hippocampal slices. We observed that compared with control mice, depression-like mice had significant reductions in spatial working memory and contextual fear memory. The level of NR2B, LTP and NMDA receptor-mediated EPSCs of depression-like mice were decreased. Ketamine treatment attenuated the memory impairment, and increased the density of NR2B and the amplitude of LTP and NMDA receptor-mediated EPSCs in the hippocampus of depression-like mice. In conclusion, depression-like mice have deficits in working memory and contextual fear memory. The decrease of NR2B, LTP induction and NMDA receptor-mediated EPSCs in the hippocampus may be involved in this process. Ketamine can improve expression of NR2B, LTP induction and NMDA receptor-mediated EPSCs in the hippocampus of depression-like mice, which might be part of the reason why ketamine can alleviate the memory dysfunction induced by depression.

## Introduction

Depression is one of the most common mental disorders which are associated with high morbidity and mortality (Ménard et al., 2016). People suffering from depression are always seen in disrupted mood, altered sleep and memory dysfunction (Benson et al., 2015). In recent years, ketamine has been demonstrated to relieve depressive symptoms quickly (Blier et al., 2012; Murrough, 2012). Despite extensive research, the mechanisms underlying how depression induces memory dysfunction and how ketamine relieves depressive symptoms remain poorly understood.

Synaptic plasticity is the biological process by which specific patterns of synaptic activity result in changes in synaptic strength, and is thought to contribute to learning and memory (Citri and Malenka, 2008). The effects of depression on synaptic activity involve multiple brain regions, such as the hippocampus, prefrontal cortex (PFC) and amygdale (Liu et al., 2017). The hippocampus is one of most commonly studied brain regions in depression research. First, the hippocampus is part of the limbic system and develops nerve fiber connectivity with emotion-related brain regions, such as amygdala. Second, the hippocampus regulates the hypothalamus-pituitary-adrenal (HPA) axis, which makes it more susceptible to stress and depression (Anacker et al., 2013). Third, it is widely believed that the hippocampus plays a key role in memory encoding and retrieving in central nervous system, and synaptic plasticity in the hippocampus is important for memory (Neves et al., 2008). Preclinical and clinical studies show that depression reduces the size of the hippocampus, and decreases the number of neuronal synapses in hippocampus (Sousa et al., 2000). The changes of synaptic plasticity in the hippocampus may be a possible reason why depression induces memory dysfunction (Duman and Aghajanian, 2012). Long-term potentiation (LTP) and long-term depression (LTD), which indicate the functional indices of synaptic plasticity, are important for memory consolidation and recall (Quan et al., 2010). So the alternations of LTP and LTD in the hippocampus may elucidate the possible mechanism underlying how depression induces memory dysfunction.

It is now well established that dysfunction of the glutamatergic system contributes to the pathophysiology of depression (Sanacora et al., 2008). On the other hand, several glutamate modulating reagents have been used to attenuate the behavioral and molecular alterations presented of depression (Calabrese et al., 2012). N-methyl-D-aspartate receptor (NMDAR) is essential in the fast synaptic glutamate neurotransmission, and ketamine, which is a noncompetitive NMDAR antagonist, shows promise as a novel treatment for depression (Cull-Candy et al., 2001). Tizabi et al. (2012) showed that although ketamine treatment did not result in significant decrease in NMDAR density in the hippocampus of depression-like rats, it induced a significant increase in AMPA/NMDA receptor density ratio in the hippocampus of depression-like rats. NMDAR consists of two obligatory NR1 subunits and two regulatory subunits, usually a combination of NR2A and NR2B (Yashiro and Philpot, 2008). Based on the above results, we guess that function of NMDAR and levels of protein expression of NMDAR subunits in hippocampus may play a role in memory dysfunction induced by depression and in antidepressant effect of ketamine.

In this study, we aimed to investigate the possible mechanism underlying how depression induces memory dysfunction and how ketamine relieves depressive symptoms. To address the issue, we compared three groups of male C57BL/6J mice: mice exposed to chronic social defeat stress (CSDS) to induce a depression-like phenotype (depression-like mice), depression-like mice treated with ketamine (depression-like mice with ketamine), and control mice that were not exposed to CSDS or treated with ketamine. Spatial working memory and long term memory were assessed by spontaneous alternation Y-maze and fear conditioning tests, respectively. We used western blot to analyze the density of NMDAR subunits in the hippocampus. We recorded LTP and NMDA receptor-mediated excitatory postsynaptic currents (EPSCs) in hippocampal slices.

## Materials and Methods

### Experiment Animals

All experimental procedures were conducted in accordance with the institutional ethical guidelines for animal experiments of China, and were approved by Animal Care and Use Committee of Jilin University. The male C57BL/6J mice (8 weeks) and CD-1 mice (18–20 weeks) were housed in a humidity- and temperature-controlled room on a 12-h and 12-h light/dark cycle. Mice accessed to water and food easily.

### Chronic Social Defeat Stress Paradigm

An extensive body of experimental and clinical evidence shows that there is a relationship between stress and depression (Moriam and Sobhani, 2013; Warren et al., 2014). The CSDS is considered to be a suitable model for production of depression-like conditions in laboratory rodents (Krishnan et al., 2007; Golden et al., 2011; Yu et al., 2011). In the CSDS model, 8-week-old C57BL/6 male mice, which were regarded as “intruder” mice, were defeated by resident CD-1 mice. The protocol was previously described (Golden et al., 2011). Briefly, C57BL/6J mouse was defeated by a larger CD-1 mouse. Although each individual defeat lasted 5 min, the defeated mouse was subjected to continuous psychological stress from CD-1 mouse through a clear perforated divider in a shared home cage for the remaining 24 h. The C57BL/6 mice were rotated daily while the CD-1 mice were not rotated during the 10-day defeat procedure. In order to select the depression-like mice, 5 min social interaction was recorded on the next day after the last attack of defeat stress. The test was composed of two parts: with and without the target CD-1 mouse in the perforated plastic box. The movements of C57BL/6 mice were recorded with a video recorder. The social interaction ratio is a ratio calculated when CD-1 is present and absent in the interaction zone. In general, the mouse, whose social interaction ratio was <1, had been regarded as depression-like mice (Krishnan et al., 2007). The mouse, whose social interaction ratio was >1, was classified as resilient. In our experiment, approximately 25% of mice showed a resilient phenotype. The mice, which were regarded as “resilient phenotype,” were not used for any further tests.

The selected depression-like mice were divided into two groups at random: depression-like mice and depression-like mice with ketamine. We selected to test a dose of ketamine of 5 mg/kg, which emerged as an average dose of ketamine displaying antidepressant-like properties in rodent behavioral paradigms in different studies (Maeng et al., 2008; Franceschelli et al., 2015). Depression-like mice with ketamine received a one-time intraperitoneal (i.p.) injection of ketamine (5 mg/kg), and depression-like mice received an injection of same amount of saline. The control C57BL/6J mice were housed in the same defeat boxes with one mouse per side of the perforated divider. The control mice were rotated every day similar to the mice undergoing defeat, but they were not permitted to contact with CD-1 mouse. Control mice were received a single i.p. injection of saline on the same day when depression-like mice were treated with saline.

**Figure 1:**
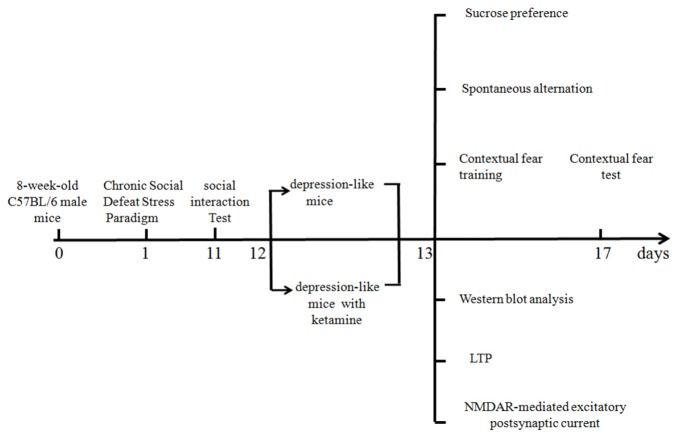
Timetable of the experimental design.

A timetable of different experiments is shown in Figure 1. Different mice were used for behavioral and electrophysiological experiments. All the experiments were tested 1 day after ketamine or saline treatments.

### Behavioral Studies

#### Sucrose Preference

Thirty-six mice were used to test sucrose preference (control *n* = 12, depression-like mice *n* = 12, and depression-like mice with ketamine *n* = 12). The mice were euthanized after test. Sucrose preference was performed as previously described (Torres-Berrío et al., 2017). Mice were accustomed to drinking water from two bottles with sipper tops for two consecutive days. From the third day, mice were permitted to have a free choice between sucrose water (1% concentration) and regular water for four consecutive days. And the position of the bottles was interchanged after each daily experiment. Sucrose preference was estimated by dividing the volume of consumed sucrose by the volume of consumed sucrose + consumed water. The average of sucrose preference of the four test days was calculated to estimate the total preference for sucrose.

#### Spontaneous Alternation in the Y-Maze Test

Thirty-six mice were used to test spontaneous alternation performance (control *n* = 12, depression-like mice *n* = 12 and depression-like mice with ketamine *n* = 12). The mice were euthanized after test. Spontaneous alternation in the Y-maze test, which was used to assess spatial working memory of mice, was performed as previously described (Satoh et al., 2007). The symmetrical Y maze consisted of three arms which were 30 cm long, 15 cm high and 8 cm wide. At the beginning of the experiment, each mouse, which was placed in the center of the Y-maze, was permitted to explore freely through the maze for 8 min. The total number of arms mice entered and the sequence were recorded. The percentage of alternation, which was the number of triads containing entries into all three arms divided by the maximum possible number of alternations, was calculated.

#### Contextual Fear Conditioning

Thirty-six mice were used to test contextual fear conditioning performance (control *n* = 12, depression-like mice *n* = 12 and depression-like mice with ketamine *n* = 12). The mice were euthanized after test. The protocol was previously described by Frankland et al. (2001). Contextual fear conditioning consisted of one training session and a test session. During training section, mice were placed in the conditioning chamber for 7 min. After the first 2 min, mice were presented with five unsignaled foot-shocks (2 s duration, 0.75 mA, 1 min apart). After training, the mice were sent back to their home cage. During testing section, the mice were placed back in the conditioning chamber for 120 s. Freezing time and activity time were recorded by video camera. The percent of freezing was used to assess contextual fear conditioning memory.

### Western Blot Analysis

Thirty-six mice were used for western blot analysis (control *n* = 12, depression-like mice *n* = 12, and depression-like mice with ketamine *n* = 12). The mice were euthanized after test. Western blotting was used to evaluate the density of NR1, NR2A and NR2B subunits on the membrane of hippocampus neurons. Western blot analysis was performed as previously described (Ma et al., 2014). Briefly, the hippocampus was homogenized in lysis buffer A (contained 250 mM sucrose, 50 mM KCl, 20 mM HEPES, 2 mM EGTA and protease inhibitors cocktails). After the lysates were centrifuged at 800 *g* for 10 min, supernatants were collected. The supernatants were centrifuged at 100,000 *g* for 60 min, and the membrane pellets were collected. The membrane pellets were dissolved in lysis buffer B (contained 150 mM KCl, 20 mM HEPES (pH 7.0), 2 mM EGTA, 1% (w/v) CHAPSO and protease inhibitors cocktails), and were incubated at 4°C for 60 min. After the soluble membranes were centrifuged at 100,000 *g* for 60 min, the supernatants were collected. The proteins, which were separated by sodium dodecyl sulfate polyacrylamide gel electrophoresis (SDS-PAGE), were transferred to polyvinylidene fluoride membranes. After washing, the membranes were blocked with non-fat skim milk, and then were incubated with appropriate primary antibody (NR2A 1:1,000, NR2B 1:1,000, and NMDAR 1:1,000, Cell Signaling Technology, Danvers, MA, USA Inc. β-actin 1:5,000, Sigma). After three times washing, membranes were incubated with the secondary antibody. Visualized by an ECL system, the membranes were developed on Hyperfilm (Amersham). The relative expression levels of all proteins were determined by densitometry and normalized to β-actin.

### Brain Slice Preparation

Thirty-six mice were used for LTP and NMDAR-EPSC (control *n* = 12, depression-like mice *n* = 12, and depression-like mice with ketamine *n* = 12). The hippocampal slices of mice were prepared as previously described (Shen et al., 2010). Briefly, mice were anesthetized with isoflurane and decapitated. The brains were rapidly removed from the cranial cavity and submerged in ice-cold artificial cerebrospinal fluid (aCSF) saturated with a mixture gas of 95%O_2_+5%CO_2_. The aCSF contained (in mM): NaCl 124, NaHCO_3_ 26, glucose 10, KCl 5, CaCl_2_ 2, MgSO_4_ 2 and NaH_2_PO_4_ 1.25, pH = 7.4. The hippocampi were dissected free, and dorsal hippocampal slices (400 μm) were sectioned by a vibrating tissue slicer (Vibratome 3000, Vibratome, USA). The slices were incubated in oxygenated aCSF at 32°C for at least 60 min before the electrophysiological recording.

### Long Term Potentiation (LTP)

The strength of synaptic transmission was quantitatively analyzed by the initial slope of the rising phase of the field excitatory postsynaptic potentials (fEPSPs). The procedure of recording fEPSP was previously described by Sarabdjitsingh et al. (2016). Briefly, bipolar stimulation electrodes (60-μm stainless steel wires insulated except for the tip) were placed on the Schaffer collaterals, and glass recording electrodes (filled with buffer, 2–5 MΩ) were placed in the CA1 stratum radiatum. At the start of each experiment, an input-output curve was established to record the slope of the fEPSP, from which maximal and half-maximal slope as well as the corresponding maximal and half-maximal stimulus intensity were determined. The half maximal stimulus intensity that was thus calculated was used throughout the remainder of the recording session. The stable baseline was maintained for at least 30 min. Then, a high frequency stimulus (HFS; 100 pulses delivered at 100 Hz) was applied to the Schaffer collateral pathway. Subsequent data were recorded for further 40 min keeping the same rate or intensity of the stimulus. Based on the records during the last 5 min before HFS, fEPSP slopes were normalized.

### NMDA Receptor-Mediated Excitatory Postsynaptic Current (NMDAR-EPSC)

NMDA receptor EPSCs were recorded as previously described (Shen et al., 2010; Xu et al., 2017). The recording aCSF was the same as above except that the aCSF contained 0.5 mM MgCl_2_ (instead of 2 mM). NMDA receptor EPSCs were recorded from CA1 region using a single glass pipette (3–5 MΩ) in whole cell recording. The intracellular pipette solution contained the following (in mM): KCl 5, Na_2_-phosphocreatine 5, lidocaine N-ethyl chloride 5, HEPES 10 and K-gluconate 130, pH = 7.4. The stimulating electrode was placed in the stratum radiatum. CA1 pyramidal cells were voltage-clamed at −70 mV. Evoked by low frequency stimulation (0.05Hz), evoked EPSCs were recorded at −70 mV holding potential in aCSF perfusion containing bicuculline (10 μmol/L) to block GABA_A_ receptor mediated inhibitory synaptic currents. NMDA receptor EPSCs were isolated at holding potential of −60 mV with NBQX (5 μM). At last, APV (50 μM) was used to show that the current was generated by NMDA receptor.

### Statistics

All data were expressed as mean ± SEM. Comparisons among data were carried out by one-way ANOVA analysis followed by Newman-Keuls *post hoc* test. A value of *P* < 0.05 was considered statistically significant.

## Results

### Sucrose Preference

**Figure 2:**
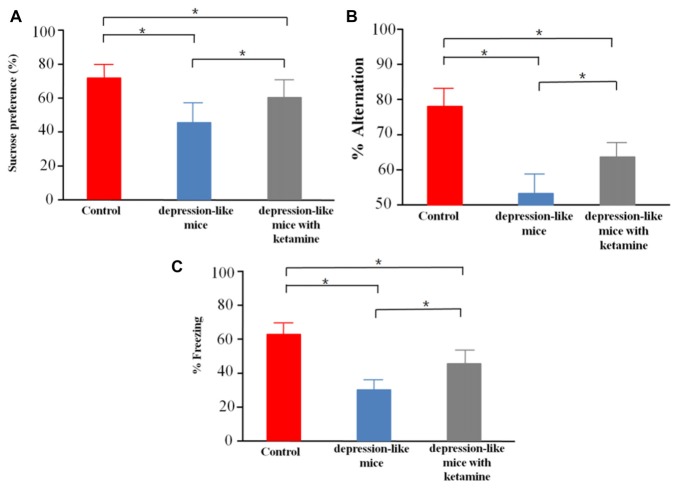
Behavioral studies of mice. **(A)** Depression-like mice had least sucrose preference among the three groups, and ketamine improved sucrose preference performance of depression-like mice. **(B)** Working memory of depression-like mice was impaired, and ketamine treatment attenuated working memory impairment of depression-like mice. **(C)** Contextual fear conditioning memory of depression-like mice was impaired, and ketamine attenuated the memory impairment of depression-like mice. Means were showed, and SEM were represented by error bars, *n* = 12, per group. *P < 0.05, one-way ANOVA, *post hoc* Tukey-Kramer.

We tested sucrose preference in control mice (*n* = 12), depression-like mice (*n* = 12), and depression-like mice with ketamine (*n* = 12). Control, depression-like mice and depression-like mice with ketamine displayed a sucrose preference over water of 75.6 ± 8.4%, 44.5 ± 10.1% and 61.8 ± 11.3%, respectively (Figure 2A). One-way ANOVA revealed significant differences among the three groups (*F*_(2,33)_ = 29.11, *P* < 0.01). *Post hoc* analysis showed that sucrose preference of depression-like mice was decreased, but ketamine ameliorated the decrease of sucrose preference in depression-like mice (*P* < 0.05: control mice vs. depression-like mice; *P* < 0.05: control mice vs. depression-like mice with ketamine; *P* < 0.05: depression-like mice with ketamine vs. depression-like mice).

### Spatial Working Memory Test in Y-Maze

Control mice, depression-like mice and depression-like mice with ketamine performed spatial working memory test with 78.4 ± 7.1%, 53.3 ± 6.7%, 65.8 ± 5.3% correct choices, respectively (Figure 2B). One-way ANOVA revealed differences among the three groups (*F*_(2,33)_ = 45.95, *P* < 0.01). *Post hoc* analysis showed that spatial working memory of depression-like mice was reduced, but ketamine ameliorated the decrease of spatial working memory in depression-like mice (*P* < 0.05: control mice vs. depression-like mice; *P* < 0.05: control mice vs. depression-like mice with ketamine; *P* < 0.05: depression-like mice with ketamine vs. depression-like mice).

### Contextual Fear Conditioning

We tested contextual fear conditioning performance in depression-like mice (*n* = 12), depression-like mice with ketamine (*n* = 12) and control mice (*n* = 12). Control, depression-like mice and depression-like mice with ketamine performed contextual fear memory test with 62.5 ± 7.7%, 35.1 ± 6.1% and 48.7 ± 8.3% of freezing, respectively (Figure 2C). One-way ANOVA revealed significant differences among the three groups (*F*_(2,33)_ = 40.85, *P* < 0.01). *Post hoc* analysis showed that contextual fear conditioning of depression-like mice was reduced, but ketamine ameliorated the decrease of Contextual fear conditioning in depression-like mice (*P* < 0.05: control mice vs. depression-like mice; *P* < 0.05: control mice vs. depression-like mice with ketamine; *P* < 0.05: depression-like mice with ketamine vs. depression-like mice).

### Membrane Protein Expression of NMDAR in Mouse Hippocampus

**Figure 3:**
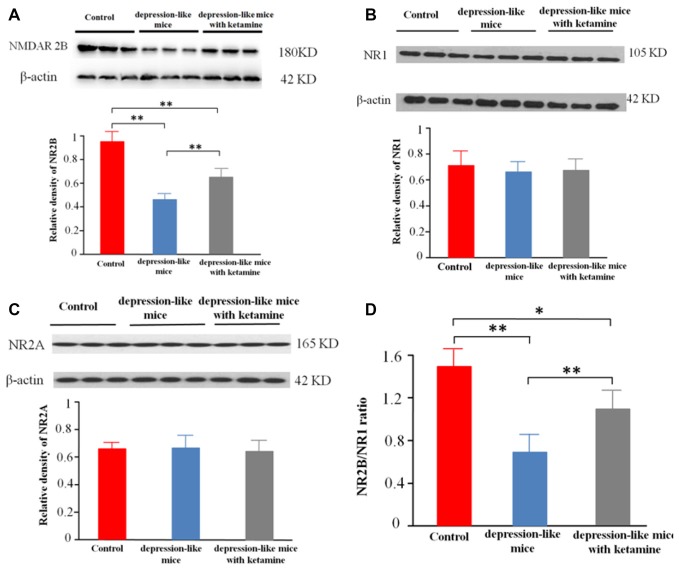
**(A)** Expression of NR2B subunit on the membrane of hippocampus neurons was tested by western blot. β-actin was selected as an internal standard and control for protein loading. **(B,C)** Expression of NR1 and NR2A on the membrane of hippocampus neurons was shown by western blot. β-actin was selected as an internal standard and control for protein loading. **(D)** NR2B/NR1 ratio. Means were showed, and SEM were shown by error bars, *n* = 12, per group. **P < 0.01, *P < 0.05, one-way ANOVA, *post hoc* Tukey-Kramer.

Significant differences were encountered with protein expression of NR2B on total hippocampal membranes among the three groups (*p* < 0.01). An additional *post hoc* analysis showed that density of NMDA receptor 2B subunit in depression-like mice was reduced compared with that of control mice (*p* < 0.01). The density of NMDA receptor 2B subunit displayed by depression-like mice treated with ketamine was lower compared with that of control mice (*p* < 0.01), but increased compared with that of depression-like mice (*p* < 0.01; Figures 3A,D). Additionally, there was no difference detected among the three groups for the densities of NR1, NR2A subunits in the hippocampus (*P* > 0.05; Figures 3B,C).

### Long Term Potentiation (LTP)

**Figure 4:**
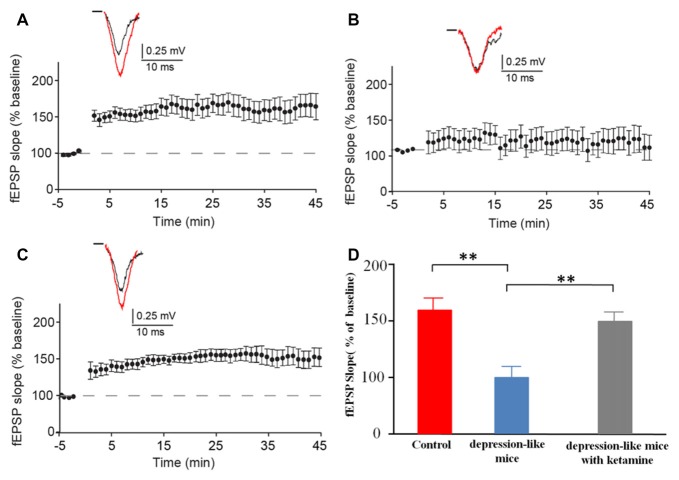
The long-term potentiation (LTP) in the hippocampal Schaffer collateral-CA1 in control mice, depression-like mice, and depression-like mice with ketamine. LTP of depression-like mice was blocked, and ketamine treatment attenuated the blockage of LTP in depression-like mice. **(A)** Control mice. **(B)** Depression-like mice. **(C)** Depression-like mice with ketamine. **(D)** Summarized data of average slope change (normalized to baseline value) from three groups of mice. Means were showed, and SEM were shown by error bars, *n* = 12, per group. **P < 0.01, one-way ANOVA, *post hoc* Tukey-Kramer.

We recorded evoked fEPSP in the stratum radiatum of the CA1 region in depression-like mice (12 slices from 12 mice), depression-like mice with ketamine (12 slices from 12 mice) and control mice (12 slices from 12 mice). High-frequency stimulation induced stable LTP in control mice and depression-like mice with ketamine. LTP was significantly blocked in depression-like mice (Figures 4A–C). There was no difference between control mice and depression-like mice with ketamine (*P* > 0.05). Figure 4D summarized the change of fEPSP slope for each group.

### NMDAR-Mediated Excitatory Postsynaptic Current (NMDAR-EPSC)

**Figure 5:**
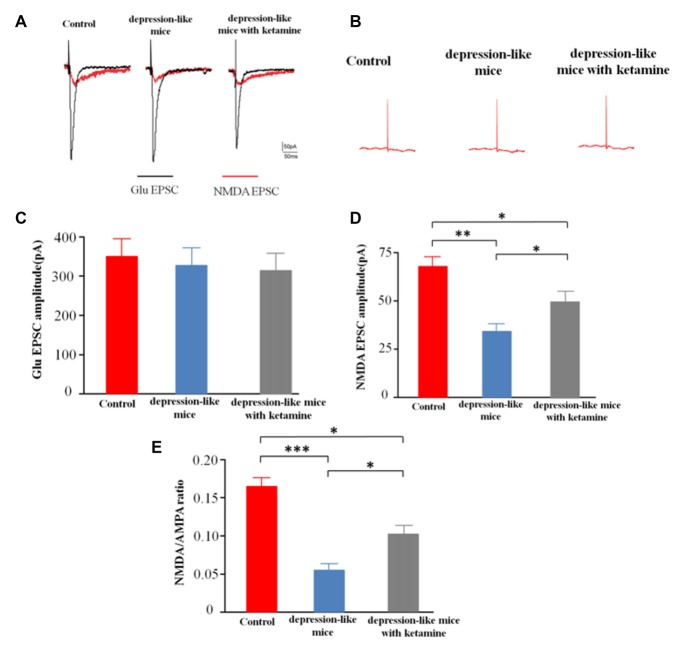
Evoked N-methyl-D-aspartate receptor (NMDAR)-mediated excitatory postsynaptic currents (EPSCs) recorded from CA1 pyramidal cells in mice brain slices. **(A)** Evoke NMDA receptor-mediated EPSC of one mouse from each group. **(B)** EPSC after treatment with APV+NBQX. **(C)** Summarized amplitude of glutamate EPSC of three groups. **(D)** Summarized amplitude of NMDA EPSCs of three groups. **(E)** Summarized NMDA/AMPA ratio of three groups. Means were showed, and SEM were shown by error bars, *n* = 12 slices from 12 mice, per group. ***P < 0.001, **P < 0.01, *P < 0.05, one-way ANOVA, *post hoc* Tukey-Kramer.

After blocking GABARs and AMPARs, whole-cell voltage clamp techniques were used to record evoked NMDA receptor-mediated EPSCs from CA1 pyramidal cells in depression-like mice (12 slices from 12 mice), depression-like mice with ketamine (12 slices from 12 mice) and control mice (12 slices from 12 mice). One-way ANOVA revealed differences of amplitudes of NMDA current among the three groups (*F*_(2,33)_ = 14.7, *P* < 0.01). *Post hoc* analysis showed that NMDAR EPSC amplitude of depression-like mice was decreased, but ketamine ameliorated the decrease of NMDAR EPSC amplitude of depression-like mice (*P* < 0.01: control mice vs. depression-like mice; *P* < 0.05: control mice vs. depression-like mice with ketamine; *P* < 0.05: depression-like mice with ketamine vs. depression-like mice). After blockade of GABAR, the ratio of NMDA/AMPA receptor-mediated currents was reduced in depression-like mice compared with those of the other two groups (*F*_(2,33)_ = 13.9, *P* < 0.01; Newman-Keuls *post hoc* test, *P* < 0.001: control mice vs. depression-like mice; *P* < 0.05: control mice vs. depression-like mice with ketamine; *P* < 0.05: depression-like mice with ketamine vs. depression-like mice; Figure 5).

## Discussion

In this study, we observed that depression-like mice had significant reductions in spatial working memory and contextual fear conditioning memory. The density of NR2B subunit in depression-like mice hippocampus was significantly decreased. Meanwhile, LTP and NMDA receptor-mediated EPSCs in hippocampal slices of depression-like mice were also reduced. Ketamine treatment mitigated the memory impairment of depression-like mice, and ketamine partially restored the levels of NR2B, LTP induction, and NMDAR receptor-mediated EPSCs in hippocampal slices of depression-like mice.

Our work showed that ketamine afforded an antidepressant-like effect in mice in sucrose preference test. Apart from our findings, many preclinical experiments have indicated that ketamine has antidepressant effects in several species and on different behavioral tests. Sałat et al. (2015) and Kara et al. (2017) demonstrated that ketamine reduced immobility of depression-like mice in the forced swim test). Garcia et al. (2008) also reported that ketamine induced antidepressant-like effect in the rat forced swimming test. Maeng et al. (2008) showed that ketamine caused antidepressant-like effects on mice in passive avoidance test. Li et al. (2011) reported that ketamine rapidly ameliorated depression-induced behavioral deficits in novelty-suppressed feeding test. Although ketamine has been reported to have an antidepressant-like effect, there are some reports of inducing depression. Ram et al. (2013) found that the combination of prepubertal onset of chronic stress and ketamine may serve as a valid novel animal model for schizophrenia-like symptoms. The antidepressant effect of ketamine was demonstrated not only in depression-like animals but also in patients. Phelps et al. (2009) and Zarate et al. (2006) demonstrated a rapid antidepressant effect could be achieved with ketamine in patients with major depression. What’s more, the antidepressant effects can persist for several days (Liebrenz et al., 2007) to even weeks (Irwin and Iglewicz, 2010; Mathew et al., 2010). In our article, we also showed that ketamine mitigated spatial working memory deficits and contextual fear conditioning memory deficits in depression-like mice. Consistent with our study, Zhu et al. (2015) showed that ketamine effectively attenuated memory impairment of depression-like rats. Besides, the effect of ketamine on memory in depressive patients was reported. Chen et al. (2017) investigated the effects of low doses of ketamine on learning and memory in patients, and found that ketamine attenuated learning and memory impairment.

The hippocampus is one of brain regions susceptible to depression. Many previous studies have shown that the hippocampus is indispensable to proper working memory (Eichenbaum, 2004; Chudasama and Robbins, 2006) and contextual fear memory (Han et al., 2016). Schaffer collaterals are axon collaterals given off by CA3 pyramidal cells in the hippocampus, and transform information from CA3 region to CA1 region. In our article, LTP in the CA1 region of depression-like mice was impaired. Consistent with our study, She et al. (2015) reported that tetanic stimulation failed to induce LTP in Schaffer collateral-CA1 synapses in depression-like rats. Besides, accumulated evidence demonstrated the alterations of LTP in Schaffer fibers-CA1 pyramid synapses in different models of depressive-like rodents. Kaster et al. (2015) reported that the amplitude of LTP in hippocampal slice was reduced in depression-like mice induced by chronic unpredictable stress. Hopeless mice, a bred-based model of depression, displayed an aberrant LTP amplitude in the hippocampus (Machado et al., 2017). Maternal separation, which was an early life stress model, impaired hippocampal LTP (Batalha et al., 2013). However, depending upon the brain area, stress influences LTP differently. For example, chronic psychological stress impairs LTP induction in hippocampal area CA1, while it has no effect on dentate gyrus LTP (Gerges et al., 2001). Our results demonstrate that ketamine attenuates the LTP impairment in CA1 region of depression-like mice. Ketamine enhances hippocampal synaptic plasticity not only in depression-like animals but also in nromal animals. Graef et al. (2015) showed that Ketamine enhanced LTP in rat hippocampus 24 h after a single i.p. treatment. Taken together, these studies support a hypothesis that altered hippocampal synaptic plasticity underlie the pathophysiology and treatment of depression.

NMDA receptor is essential for excitatory and inhibitory synaptic transmission (Cull-Candy et al., 2001; Hansen et al., 2017). Moreover, NMDA receptor-dependent synaptic plasticity in the hippocampus has been supposed to be the key mechanism of memory and learning (Zhuo, 2009). We found out that expression of NMDA receptor 2B subunit in the hippocampus of depression-like mice was decreased. On the contrary, Calabrese et al. (2012) demonstrated that chronic stress increases the expression of NR1 and NR2B in rat hippocampus. The following factors might contribute to the differences. Both the experimental animals and depression-like models were different. In our study, the NMDA receptor-mediated EPSCs in hippocampus CA1 region from depression-like mice were decreased. Yuen did the same kind of research in PFC. Yuen et al. (2012) demonstrated that NMDA EPSCs in PFC pyramidal neurons from repeatedly stressed rats were markedly reduced, which caused the detrimental effect on PFC-dependent memory impairment. So the decrease of NMDAR EPSC and the density of NR2B in the hippocampus play a role in memory dysfunction induced by depression. Ketamine attenuates the decrease of NMDAR EPSC and the density of NR2B in the hippocampus, which might underlie the ability why ketamine can alleviate the memory dysfunction induced by depression. Duman and Li (2012) demonstrated another possible mechanism by which ketamine relieves depressive symptoms. Ketamine increases brain-derived neurotrophic factor (BDNF) release and activates mammalian target of rapamycin (mTOR) signaling, which then increases synthesis of synaptic proteins. These effects of ketamine reverse the atrophy of neurons caused by depression.

There are a number of limitations associated with our study. The major limitation of the experimental design is the lack of a control group treated with ketamine. However, recent studies, which have shown the impact of ketamine on hippocampal synaptic transmission and plasticity in the hippocampus of normal rodents, may help justify this limitation. Ribeiro et al. (2014b) reported that the direct application of ketamine to mouse hippocampal slices reduced LTP. But the decrease of LTP was not persistent. Ribeiro et al. (2014a) also showed that a single i.p. injection of ketamine did not affect LTP 24 h later in adult male C57BL/6 mice. The second limitation of this study was that we only tested LTP in Schaffer collaterals→CA1 pathway of the hippocampus. There are several important pathways which have been shown to sustain LTP in the hippocampus (such as Entorhinal cortex→dentate gyrus, Mossy fibers→CA3, Schaffer collaterals→CA1 and CA1→subiculum; Lynch, 2004). In our article, we showed the alternations of LTP in Schaffer collaterals→CA1 pathway, but we did not test synaptic plasticity in other pathways in the hippocampus. The third limitation was that we didn’t test the LTP in PFC of depression-like mice. Depression results in structural alterations (including regulation of neurogenesis, dendrite length and spine density) in hippocampus and PFC (Duman and Aghajanian, 2012). Many previous articles showed that the PFC plays a role in the ketamine’s antidepressant actions. For example, Duman and Li (2012) showed that ketamine causes a rapid induction of synaptogenesis and spine formation in the PFC. In our future work, we will test the LTP and NMDA receptor-mediated EPSC in PFC of depression-like animals.

In conclusion, depression-like mice have deficits in working memory and contextual fear memory. The decrease of NR2B, LTP induction, and NMDA receptor-mediated EPSCs in the hippocampus may be involved in this process. Ketamine can improve the density of NR2B, LTP induction, and NMDA receptor-mediated EPSCs in the hippocampus of depression-like mice, which is part of the reason why ketamine can alleviate the memory dysfunction induced by depression.

## Author Contributions

LS designed the study. YY, WJ and HZ performed the experiments and developed the data analysis. All of the authors discussed the data and co-wrote the article.

## Conflict of Interest Statement

The authors declare that the research was conducted in the absence of any commercial or financial relationships that could be construed as a potential conflict of interest.

## Supplementary Material

The Supplementary Material (Supplementary Data Sheet 1) for this article can be found online at: https://www.frontiersin.org/articles/10.3389/fnbeh.2018.00229/full#supplementary-material
