# Supplementary material for: Effect of Ketamine on LTP and NMDAR EPSC in Hippocampus of the Chronic Social Defeat Stress Mice Model of Depression
Source: Front Behav Neurosci. 2018 Oct 9;12:229. doi: 10.3389/fnbeh.2018.00229 (PMC6189398; doi:10.3389/fnbeh.2018.00229)
Supplement: Supplementary file 1 [file Data_Sheet_1.PDF]

Raw data of the behavioral tests

Sucrose preference

Control (%)	depression-like mice (%)	depression-like mice with ketamine (%)
73.1276	41.5273	58.4741
71.3295	39.3653	56.0552
68.6324	36.1223	52.4269
70.4305	38.2843	54.8457
83.9162	54.4992	72.9873
77.6229	46.9322	64.5212
94.7048	67.4712	87.5005
70.4305	38.2843	54.8457
63.2381	29.6363	45.1703
74.0267	42.6083	59.6835
83.0172	53.4182	71.7778
76.7238	45.8512	63.3118

Spatial working memory test in Y-maze

control (%)	depression-like mice (%)	depression-like mice with ketamine (%)
76.3102	51.3280	64.2400
74.7904	49.8938	63.1055
72.5107	47.7425	61.4038
74.0305	49.1767	62.5383
85.4292	59.9332	71.0471
80.1098	54.9135	67.0763
94.5481	68.5383	77.8542
74.0305	49.1767	62.5383
67.9512	43.4399	58.0002
77.0702	52.0451	64.8073
84.6693	59.2161	70.4799
79.3499	54.1964	66.5091

Contextual fear conditioning

control (%)	depression-like mice (%)	depression-like mice with ketamine (%)
60.2336	33.3046	46.2570
58.5854	31.9988	44.4804
56.1130	30.0402	41.8153
57.7613	31.3459	43.5920
70.1232	41.1391	56.9172
64.3543	36.5690	50.6988
80.0127	48.9737	67.5774
57.7613	31.3459	43.5920
51.1682	26.1229	36.4852
61.0578	33.9575	47.1454
69.2991	40.4863	56.0289
63.5302	35.9161	49.8104

Western Blot

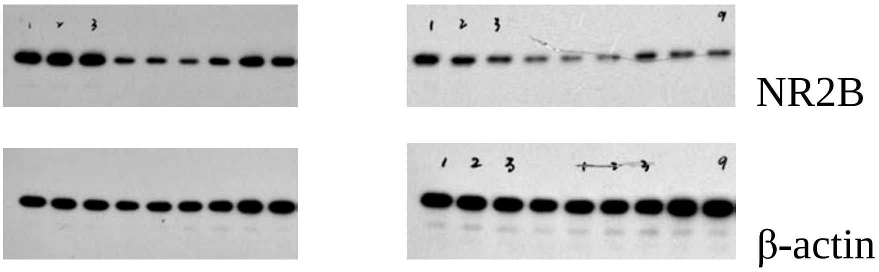

Expression of NR2B subunit on the membrane of hippocampus neurons was tested by western blot. β-actin was selected as an internal standard and control for protein loading.

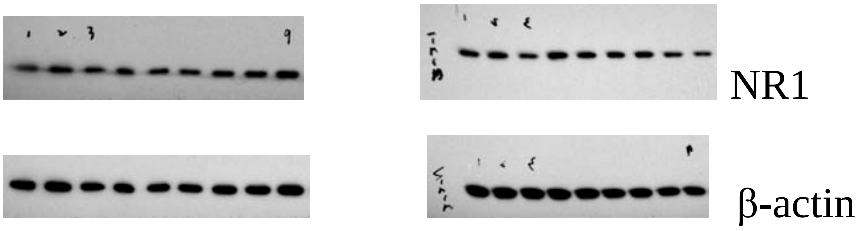

Expression of NR1 subunit on the membrane of hippocampus neurons was tested by western blot. β-actin was selected as an internal standard and control for protein loading.

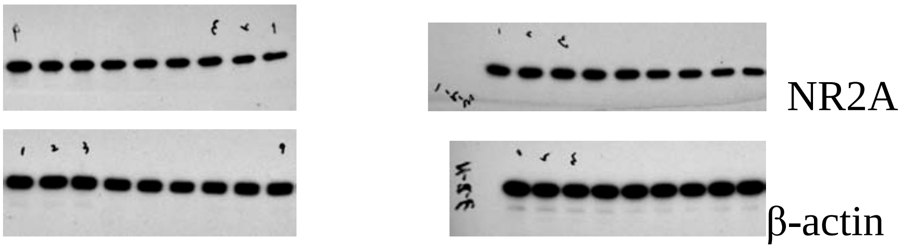

Expression of NR2A subunit on the membrane of hippocampus neurons was tested by western blot. β-actin was selected as an internal standard and control for protein loading.

LTP

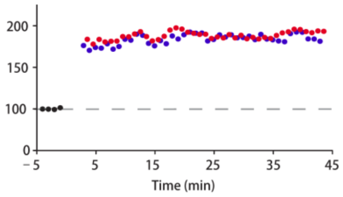

Control mice.

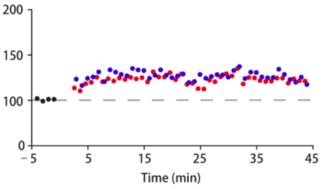

Depression-like mice.

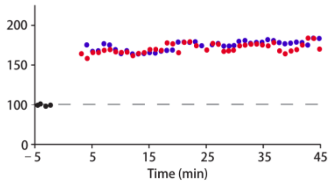

Depression-like mice with ketamine.

NMDA

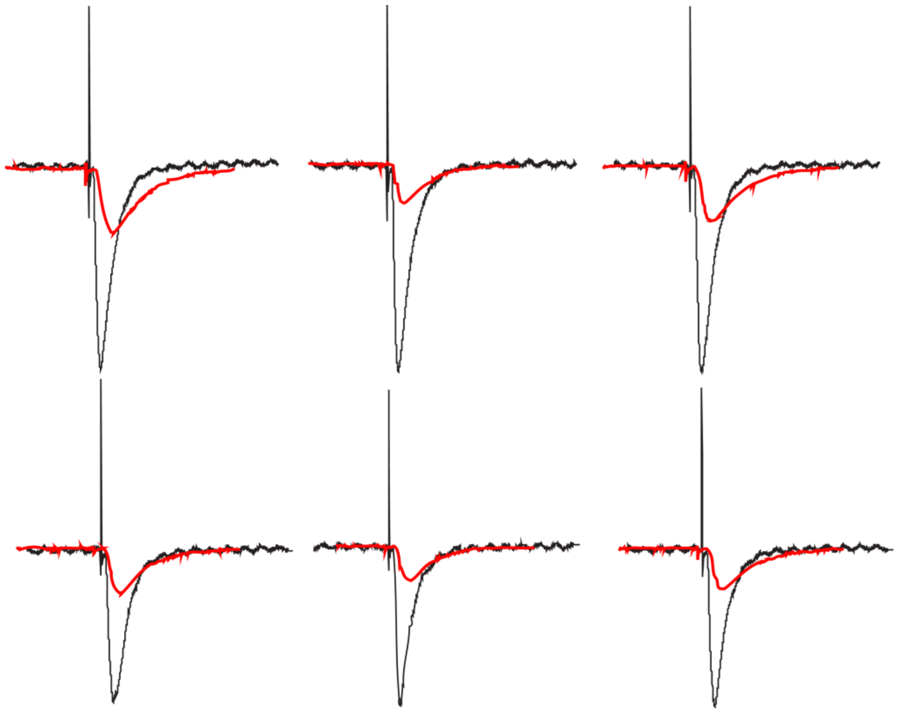

Control mice. Depression-like mice. Depression-like mice with ketamine.
